# Is there a role for surgery in the management of isolated secundum atrial septal defect in adults?

**DOI:** 10.5830/CVJA-2014-015

**Published:** 2014-06

**Authors:** Cengiz Bolcal, Gokhan Arslan, Murat Kadan, Suat Doganci, Mehmet Arslan, Cem Barcin, Atilla Iyisoy, Vedat Yildirim

**Affiliations:** Department of Cardiovascular Surgery, Gulhane Military Academy of Medicine, Ankara, Turkey; Department of Cardiovascular Surgery, Gulhane Military Academy of Medicine, Ankara, Turkey; Department of Cardiovascular Surgery, Gulhane Military Academy of Medicine, Ankara, Turkey; Department of Cardiovascular Surgery, Gulhane Military Academy of Medicine, Ankara, Turkey; Department of Cardiovascular Surgery, Gulhane Military Academy of Medicine, Ankara, Turkey; Department of Cardiology, Gulhane Military Academy of Medicine, Ankara, Turkey; Department of Cardiology, Gulhane Military Academy of Medicine, Ankara, Turkey; Department of Anesthesiology, Gulhane Military Academy of Medicine, Ankara, Turkey

**Keywords:** atrial septal defect, device occluder, cardiac surgery, adult congenital heart disease

## Abstract

**Objectives:**

The aim of this retrospective study was to compare the short-term outcomes of surgical versus transcatheter closure of secundum atrial septal defect (ASD) in adults.

**Methods:**

From January 2008 to October 2012, 229 patients aged 18 years and older with significant isolated secundum ASDs were admitted to our hospital. We focused only on objective data obtained from their medical records. We collected and compared a total of 163 patients with isolated secundum ASD, who were treated with device occlusion or surgical closure, and had no missing data. Postoperative outcomes, rhythm disturbances, residual ASD, infection rates and length of hospital stay were compared.

**Results:**

Complete follow-up data were available for 42 (46%) patients in the device group and for 121 (87%) in the surgery group. Complete closure was observed in 41 of the 42 patients (97.6%) in the device group (*p* = 0.258) and in all 121 in the surgery group (100 %) (*p* > 0.05). There were no mortalities. The mean length of hospital stay in the device group was 1.92 ± 0.43 days and in the surgery group 7.14 ± 0.14 days (*p* < 0.01).

**Conclusions:**

The transcatheter approach for closure of ASDs is an effective and safe treatment option when performed for certain indications. Broadening the spectrum of indications may cause some adverse events. Surgical treatment remains a good alternative for all patients with ASDs and can be performed safely in order not to increase procedure-related complications.

## Abstract

Isolated atrial septal defect (ASD) is the most common form of congenital heart abnormalities in adults and approximately 80% are located in the region of the fossa ovalis (ostium secundum ASD).^1^ Indications for closure are in cases where the ratio of pulmonary-to-systemic flow (Qp/Qs) is higher than 1.5, without significant elevation of pulmonary vascular resistance.

Surgical closure of ASDs has been performed for over 60 years and techniques have steadily improved, using smaller incisions and minimally invasive techniques. On the other hand, in the last 20 years, various transcatheter ASD closure techniques and devices have been developed, among which, percutaneous treatment with a septal occluder device is the most popular.^2^,^3^

Despite increasing use of occluder devices and the fact that studies have been published internationally pointing out some of the advantages and disadvantages compared with surgery in adults, no formal comparison of efficacy, morbidity and complications has been published.^2^-^4^ We present a retrospective comparison of short-term (three months) results for transcatheter and surgical closure of 163 ostium secundum ASD patients in a university hospital.

## Methods

This was a retrospective analysis at a single centre, studying two groups of adult patients with isolated secundum ASDs who were treated by occlusion with a device, or with surgical closure. Postoperative outcomes, rhythm disturbances, residual ASD, infection rates and length of hospital stay of these two groups were compared.

Between January 2008 and October 2012, 229 patients admitted to our centre, aged 18 years and older with significant isolated secundum ASDs, who had undergone surgical or transcatheter closure of the ASD and who had follow-up data, were assessed in this trial. Follow up was obtained from a chart review and routine three-month post-repair check-up records. We focused only on objective data obtained from the medical records and we compared 163 patients with complete data in two groups (device closure and surgery).

As routine practice in our clinic, secundum ASD assessment is performed using transthoracic echocardiography (TTE) and transoesophageal echocardiography (TEE). Defect size is estimated by TTE and TEE, and also by balloon sizing. The patients are assigned to device treatment or surgical closure according to septum morphology, location of the defect, the presence of rims around the defect, and the patient’s choice.

Our indications for device implantation are the presence of a secundum ASD (diameter ≤ 30 mm), left-to-right shunt with Qp/Qs ratio ≥ 1.5, presence of right ventricular volume overload, and symptoms associated with the defect (arrhythmias, transient ischaemic attack). On the other hand, indications for surgical repair are the presence of associated congenital cardiac anomalies requiring surgical repair (including primum ASD, sinus venosus ASD and multiple defects that would not be adequately covered by a device), pulmonary vascular resistance ≥ 7 Woods units, haemodynamic instability, intracardiac thrombi, contra-indications to antiplatelet agents, and insufficiency of the rims. The patients were fully informed on the treatment options and then decided with their cardiologists which option they would choose.

Device implantation was performed under general anaesthesia and guidance with continuous TEE monitoring. Defect diameters were measured by TEE. The appropriate-sized device was then screwed on the cable and advanced inside the correct-sized sheath (6–14 F). The sheath is usually positioned over the guide wire inside the left upper pulmonary vein or in the middle of the left atrium. The Occlutech Figulla ASD occluder (Occlutech International, Sweden, 6–40 mm, 3-mm increments) was used in all patients.

Under fluoroscopic and TEE guidance, both left and right discs were deployed in sequence across the defect. After deployment of the device, the geometry of the device and the presence of a residual shunt were evaluated. The configuration of the tissue adjacent to the device, including the ascending aorta, atrioventricular valves, pulmonary vein, superior vena cava, inferior vena cava and coronary sinus were observed. Before releasing the device, a gentle ‘Minnesota wiggle’ was performed to verify the stability of the device. Final examination by TEE was performed to verify the device location and any residual shunting.

Unfractioned heparin was administered to keep the activated clotting time (ACT) at > 250 seconds during the entire procedure. We also used appropriate antibiotics before and after the procedure.

Patients are usually observed overnight and discharged home the following day. All patients were instructed on prophylaxis for infective endocarditis for a total of six months after device placement. Aspirin 300 mg (for six months) and clopidogrel 75 mg (for three months) were initiated after closure. Before discharge, a chest X-ray, electrocardiography and echocardiography were performed.

The patients undergoing surgical treatment were operated on under general anaesthesia using the standard approach. The right atrium was opened following median sternotomy and the ASD closed by primary suture or pericardial patch under cardiopulmonary bypass. The patients were then taken to the intensive care unit, and the following day to the postoperative ward for recovery, until their condition had stabilised, after which they were discharged.

Post-procedure records up to discharge from the hospital and routine three-month results related to the end-points were compared. Three months after the procedure, all patients in the study underwent a physical examination, electrocardiogram, chest radiograph and transthoracic echocardiogram with colour Doppler.

The primary end-point was rhythm disturbances, residual ASD, infection, or any cardiovascular procedure-related major or minor complications, excluding death. The secondary end-point was death related to both procedures.

Patients were considered to have had successful ASD closure if they had no or < 2-mm-wide colour jet residual shunts as assessed by colour Doppler echocardiography. Early efficacy was successful closure of the defects by device or operation without moderate (2–4-mm-wide colour jet) or large (≥ 4-mm-wide colour jet) residual shunts, or major complications after discharge from hospital (cerebral embolism, cardiac perforation with tamponade, endocarditis, repeat operation, cardiac arrhythmias requiring permanent pacemaker placement or long-term antiarrhythmia medication, device embolisation requiring immediate surgical removal or death due to the procedure).

Safety was defined as the absence of death or complications. Minor complications included device embolisation with percutaneous retrieval, pericardial effusion requiring medical management, evidence of device-associated thrombus formation without embolisation (with or without treatment), cardiac arrhythmia with treatment, phrenic nerve injury, accesssite haematoma, other vascular access-site complications, retroperitoneal haematoma, surgical wound complications or infection.

## Statistical analysis

All the available data were analysed by the SPSS program (Statistical Package for Social Sciences for Windows 17.0) (Chicago, IL, USA). Descriptive statistical methods (number, percentage, mean, standard deviation) were used on the data. Differences in variables were analysed using the independent samples *t*-test and chi-square tests as appropriate, and *p*-values < 0.05 were considered significant.

## Results

A total of 163 patients with complete medical records from the 229 patients who were treated for secundum ASDs were registered in the study. Table 1 summarises the baseline clinical and demographic characteristics of the two groups, together with the results obtained. Mean age for the device group was 24 ± 0.35 years, whereas it was 28.46 ± 0.7 years for the surgery group. In the device group, the median defect diameter assigned to percutaneous closure was 14.5 mm (range 4–28 mm), whereas it was 25 mm (range 3–46 mm) for surgical closure.

**Table 1:** Demographic and baseline characteristics of the patients.

	*Surgery*	*Device*	p*-value*
Patients (*n*)	121	42	0.258
Male, *n* (%)	105 (87)	42 (100)	
Female, *n* (%)	16 (13)	0 (0)	
Mean age (years)	24.46 ± 0.7	24 ± 0.35	0.554
Mean EF (%)	65 ± 0.36	63.9 ± 0.43	0.108
Mean Qp/Qs	2.2 ± 0.03	1.82 ± 0.46	< 0.05
Mean PAP (mmHg)	33.8 ± 0.89	28.2 ± 1.34	< 0.05

EF, ejection fraction; PAP, pulmonary artery pressure; Qp/Qs, pulmonary-to-systemic flow ratio.

The mean pulmonary and systemic blood flow (Qp/Qs) ratio in the device group was 1.82 ± 0.46 and in the surgery group it was 2.2 ± 0.03 (*p* < 0.05). The pulmonary arterial pressure (PAP) ratio in the device group was 28.2 ± 1.34 mmHg and in the surgery group it was 33.8 ± 0.89 mmHg (*p* = 0.01). The mean length of hospital stay in the device group was 1.92 ± 0.43 days and in the surgery group, 7.14 ± 0.14 days (*p* < 0.05).

Follow up was available for 42 (46%) patients in the device group and for 121 (87%) in the surgery group. During the follow-up period, complete closure was observed in 41 of 42 patients (97.6%) in the device group and in all 121 patients in the surgery group (100%) (*p* = 0.258). In one patient (3%), a small (< 2 mm wide) colour jet residual shunt as assessed by colour Doppler echocardiography in the device group and the patient was followed up without additional processing.

Cardiac arrhythmias requiring pacemaker placement or longterm anti-arrhythmia medication were not observed. Minor cardiac arrhythmias not requiring medical treatment in hospital were observed in one patient in the device group (3%) (first-degree AV block), and in three patients in the surgery group (2%) (two with sinus arrhythmia and one with first-degree AV block) (*p* = 0.72). During the three-month follow up, arrhythmias were not observed in either group (χ^2^ = 0.0, *p* = 0.0).

The mean amount of mediastinal bleeding was 364 ± 19.6 ml, and moderate bleeding was observed in two patients (1.5%). These patients in the surgery group underwent revision surgery for bleeding. Pericardial effusion due to surgery was observed in four patients (3.3%) and treated with medical therapy. Infection of the surgical site was not observed, while pulmonary infections were observed in two patients (1.5%) in the surgery group. Haematoma in the femoral region was observed in four patients (9%) in the device group.

None of the patients experienced worsening related to mitral or tricuspid valve regurgitation, and none developed left ventricular failure in either group. There were no cases of erosions, ischaemic stroke, cardiac perforation, late embolisation, thrombus formation, or malposition of the device after percutaneous closure of the ASD. Mortality was 0% for both groups. Complications and outcomes of the patients are given in Table 2.

**Table 2:** Complications and outcomes during follow up.

	*Device*	*Surgery*	p*-value*
Residual shunt, *n* (%)	1 (2.4)	0	0.258
Moderate bleeding and revision, *n* (%)	–	2 (1.7)	
Pulmonary infection, *n* (%)	0	2 (1.7)	
Surgery site infection	–	0	
Arrhythmias in hospital, *n* (%)	1 (2.4)	3 (2.5)	0.726
Arrhythmias during follow up	0	0	
Mean hospital stay (days)	1.92 ± 0.43	7.14 ± 0.14	< 0.001*
Mean mediastinal drainage (ml)	–	364 ± 19.6	
Pericardial effusion, *n* (%)	–	4 (3.3)	
Femoral haematoma, *n* (%)	4 (9)	–	

*Independent samples *t*-test.

## Discussion

Transcatheter closure of ASD with septal occluder devices has increasingly become a practical alternative to surgical techniques in patients with suitable ASDs. Its principal benefits include fewer complications, the absence of an incision scar, shorter length of hospital stay and less discomfort for the patients. The efficacy of percutaneous device closure has been well reported from case series and comparative studies.^2^-^5^

Our study confirms previous findings that ASD closure is generally successful and involves low morbidity and mortality rates with both surgical and device closure.^5^-^8^ In some series,^6^,^7^ complete closure (with no residual shunt) was achieved less often by the percutaneous method. In our study, however, as in other series, the success rate was similar for both techniques.

Complication rates with either treatment modalities were reported to be low.^5^-^8^ The most frequently reported complications after surgical closure were arrhythmia, pericardial effusion, mediastinal bleeding needing revision, and infection, together with the potential risks associated with blood transfusion. On the other hand, device closure may be accompanied by tamponade, embolisation of the device, symptomatic gaseous embolism, or unwanted problems related to femoral puncture.^1^,^5^,^9^

In the device group, femoral haematoma was observed in four patients in our study. There was no case of thrombus-related embolism or cardiac tamponade. However complications, although minor, occurred more frequently in the surgery group. There were four cases of pericardial effusions in this group. Length of hospital stay was significantly longer in the surgery group, which is in agreement with other comparative studies.^5^-^8^ Apart from the length of hospitalisation, the groups were comparable, with good cardiac outcomes.

Another important consideration favouring device closure is the absence of a surgical scar. This is a notable advantage, particularly in female patients. Parallel with improvements in closure device technology, there have also been improvements in the surgical approach, such as using mini-sternotomy, thoracotomy, and endoscopic and robotic surgery.^10^-^12^ Although we usually use the minimally invasive approach, due to the patient numbers in our surgical group, we performed only a standard sternotomy. Our results are comparable with the device group, with good outcomes.

There were some limitations to this study. It had a retrospective design and patient groups were non-randomised. Only 71% of patients had complete sets of data. In retrospective studies, managing to collect full sets of data is difficult. Additional studies and longer-term follow up of these or other randomised patient groups would be valuable for making recommendations about treatment modalities.

## Conclusion

There have recently been dramatic developments in both surgical and percutaneous methods of treatment of ASDs, together with the development of emerging technologies. New strategies are an improvement on older methods and device closure is replacing open-heart surgery, especially in suitable patients with isolated ASDs. The range of indications for device closure is frequently not well defined. Increasing the range of indications for device closure, however, may cause complications that would make surgical treatment a more attractive option. As is evident from this study, surgical treatment can be performed efficaciously with low complication and mortality rates. The surgical approach therefore maintains its position as an essential alternative method, particularly in patients who are unsuitable for device closure.
